# The Relationship Between Learning Environment Perception, Achievement Goals, and the Undergraduate Deep Learning Approach: A Longitudinal Mediation Model

**DOI:** 10.3390/jintelligence13020019

**Published:** 2025-02-11

**Authors:** Tingzhi Han, Guoxing Xu

**Affiliations:** 1 School of Education, Jiangnan University, Wuxi 214122, China; 2 Institute of Higher Education, East China Normal University, Shanghai 200062, China

**Keywords:** learning environment perception, achievement goal, deep learning approach, longitudinal mediation model, undergraduates

## Abstract

This study examines the relationship between undergraduates’ learning environment perception, achievement goals, and the deep learning approach in a research-oriented Chinese university with a longitudinal mediation model. A total of 260 undergraduates, including 135 top-notch undergraduates and 125 ordinary undergraduates, participated in all three rounds of investigation across 18 months. The results revealed that (a) mastery-approach goals and mastery-avoidance goals can partially mediate the longitudinal impact of undergraduate students’ perception of the learning environment on their deep learning approaches, while performance-approach goals and performance-avoidance goals do not serve as longitudinal mediators in the influence of undergraduate students’ perception of the learning environment on their deep learning approaches. (b) There are two longitudinal mediating pathways, namely the mastery-approach goals and mastery-avoidance goals, in the influence process of perceived learning environment on deep learning approaches among undergraduates. (c) The mediating effect of mastery-approach goals on the influence of learning environment perception on undergraduates’ deep learning approaches is more significant than mastery-avoidance goals. The findings of this study will contribute new empirical evidence that can inform teaching reform initiatives aimed at enhancing the learning quality of undergraduates.

## 1. Introduction

The deep learning approach, characterized by high-level cognition that facilitates students’ search for connections, understanding, models, and evidence (Entwistle 2000), enables learners to engage in focused, long-term reflection and transformative thinking regarding specific problems or fields (Biggs et al. 2001). Consequently, the deep learning approach has long been recognized as a crucial indicator of learning quality. With the widespread expansion of higher education in recent years, the quality of undergraduate education in China has received significant attention, making the evaluation of undergraduate students’ learning quality a prominent research topic in the field of Chinese higher education (Han et al. 2024).

The learning environment is a crucial factor influencing students’ deep learning approaches. The learning environment comprises not only the objective features of school environments but also students’ perceived aspects, such as their perception of teaching quality (Lawless and Richardson 2002), teaching objectives (Diseth et al. 2006), assessment practices (Segers et al. 2008), and other elements that students perceive. In recent years, researchers have increasingly shifted their focus towards learners’ perceptions of the external learning environment rather than the objective characteristics of the environment itself and its impact on learning.

Empirical research has demonstrated the influence of the perceived learning environment on learners’ adoption of achievement goals, which, in turn, directly impact their graded performance, intrinsic motivation, and deep learning approach. Different types of achievement goal orientations can mediate the relationship between students’ perception of the learning environment and their learning motivation, strategy, and outcomes in various ways. Prior cross-sectional studies by Church et al. (2001) and Lau and Lee (2008) have shed light on these relationships.

While existing studies have verified the relationship between learning environment perception and students’ learning approaches, as well as the different mediating roles played by various types of achievement goals, these studies are predominantly cross-sectional. Therefore, there is a need to explore the longitudinal interactions among learning environment perception, achievement goals, and deep learning approaches. In the existing research on the correlation between undergraduate students’ learning environment perception and deep learning approach, there has been limited exploration of the longitudinal mediating role of achievement goals. Consequently, this study aims to complement the existing evidence from longitudinal studies on the relationship between undergraduate students’ learning environment perception and deep learning approach.

## 2. Literature Review

### 2.1. Deep Learning Approach

The academic exploration of the deep learning approach in education dates back to Marton and Säljö’s seminal 1976 study, which revealed a strong correlation between learners’ cognitive processes and the quality of their learning outcomes (Marton and Dahlgren 1976). Prior to defining deep learning, they investigated college students’ non-verbatim reading habits, finding that learning is not just about reproducing information but understanding the underlying meaning of stimuli (Marton 1975). In their 1976 experiment at the University of Gothenburg, involving 40 first-year female students, they found that while vocabulary varied, participants’ responses could be categorized into four distinct ways of understanding, indicating different levels of comprehension and perception of tasks. This led to the introduction of “deep” and “surface” learning processes (Marton and Säljö 1976a). They argued for a shift from assessing the quantity of knowledge to emphasizing the quality of learning outcomes.

Marton and Säljö’s subsequent experiment with 20 students showed that task-specific questions influenced students’ cognitive processing. Comprehensive questions led to deep processing, while more basic questions prompted surface-level processing (Marton and Säljö 1976b). Building on their work, Biggs, Entwistle, and Ramsden further developed the concept of deep learning. Biggs categorized learning approaches into deep, surface, and achieving approaches, defining the deep approach as focusing on a comprehensive understanding of the subject (Biggs 1993). Entwistle described deep learning as seeking meaning by drawing on prior experiences and relating facts (Entwistle 2012). Ramsden characterized it as students’ intent to understand a task in connection with other course materials or prior knowledge, integrating new information with personal experiences (Ramsden 2003).

Although different researchers have provided various conceptual definitions and descriptions of the deep learning approach based on different research perspectives, they all emphasize the significant benefits of the deep learning approach for students’ academic growth and development. Ultimately, the deep learning approach leads to an improvement in the quality of students’ learning. (Paver and Gammie 2005; Heikkilä and Lonka 2006; Clayton et al. 2010; Tarabashkina and Lietz 2011; May et al. 2012) The deep learning approach is intricately linked to students’ learning quality. Numerous scholars have empirically confirmed the positive predictive role of the deep learning approach in enhancing learners’ learning quality. Duff (2004) found that students who engaged in deep learning strategies consistently achieved better academic outcomes. Gijbels et al. (2005) classified learning outcomes into three hierarchical levels: (a)understanding a single concept, (b) understanding the interconnections between different concepts, and (c)understanding the connections between concepts and their underlying principles. Their study, conducted with law students, revealed that those who adopted deep learning strategies exhibited higher levels of learning outcomes compared to students using surface learning approaches. Moreover, Chotitham et al. (2014) conducted an analysis of deep learning practices among students at Chulalongkorn University, investigating the effects of deep learning on academic achievement. Their findings confirmed that deep learning significantly enhanced students’ academic performance, providing further evidence of the positive impact of deep learning on achievement.

### 2.2. Learning Environment Perception

The learning environment is a multifaceted construct encompassing educational, physical, social, and psychological dimensions. It includes not only the physical setting of the classroom but also the teaching methods employed, the nature of interactions with peers and instructors, and the overall atmosphere that shapes students’ educational experiences (Kennedy et al. 2013). In higher education, “Learning Environment Perception” refers to students’ subjective interpretations of various aspects of their learning environment. These perceptions reflect their overall learning experiences and are frequently used to assess the educational effectiveness of a department or institution (Ramsden 1991). As a key determinant of academic outcomes, students’ perceptions of their learning environment have been extensively studied, underscoring their critical role in shaping the learning process (Guo et al. 2023).

The most well-known tool for measuring learners’ perceptions of the learning environment is the Course Experience Questionnaire (CEQ) developed by Ramsden. The CEQ was developed during a period of significant reform in Australia’s higher education sector in the late 1980s. Following the release of the Green and White Papers, which called for greater accountability and the establishment of performance indicators for institutions, student satisfaction was highlighted as a key metric. In response, the Performance Indicators Research Group (PIRG) was tasked with identifying quantitative indicators to assess both individual institutions and the university sector as a whole. The CEQ, created by Ramsden in 1991, was designed to measure students’ perceptions of teaching quality. It focuses on capturing valid and reliable data related to the elements of teaching that students directly experience without attempting to assess every aspect of teaching effectiveness (Byrne and Flood 2003). Since 1992, the Graduate Careers Council of Australia (GCCA) has employed the Course Experience Questionnaire (CEQ) to assess students’ perceptions of their learning environment. The data collected through this instrument is rigorously analyzed and published on an annual basis. The indicator system of the CEQ has evolved in response to changes in the higher education landscape in Australia. Beginning in 2002, the GCCA established three primary dimensions to evaluate students’ course experiences: Good Teaching (Good Teaching Scale, GTS), Generic Skills (Generic Skills Scale, GSS), and Overall Satisfaction (Overall Satisfaction Item, OSI). Participation in the survey by universities is mandatory for these core dimensions. Furthermore, a set of optional dimensions is available for institutions to select, including Clear Goals and Standards Scale (CGS), Appropriate Workload Scale (AWS), Appropriate Assessment Scale (AAS), Intellectual Motivation Scale (IMS), Student Support Scale (SSS), Graduate Qualities Scale (GQS), and Learning Resources Scale (LCS). The decision to include these additional dimensions is left to the discretion of the participating universities (ACER 2002). To date, the CEQ has gained widespread adoption, not only in Australia but also across numerous countries worldwide (Thien and Ong 2016).

### 2.3. The Impact of Learning Environment Perception on Deep Learning Approach

Research on environment-related variables within educational contexts has been thoroughly explored, establishing learning environments research as a well-defined and vital field of study (Mutlu and Yıldırım 2019). Building on the foundational works of Lewin (1936) and Murray (1938), which emphasized the interaction between the environment and individual characteristics in shaping human behavior, and further guided by Getzels and Thelen’s (1960) investigation into the dynamic interplay between personality needs, expectations, and environmental influences, scholars have increasingly focused on the significance of learners’ perceptions of their learning environments. This growing area of inquiry has gained substantial attention in educational research, emphasizing its central role in shaping students’ academic experiences and outcomes (Goh and Khine 2002; Mutlu and Yıldırım 2019).

The relationship between the learner, the task, and the surrounding context is dynamic and interactive, ultimately determining how students engage with learning materials and achieve academic success (Baeten et al. 2010; Entwistle and McCune 2004). In fact, research suggests that it is students’ subjective perceptions of their learning environment, rather than the objective context itself, that more accurately predict their learning behaviors and outcomes (Asikainen and Gijbels 2017; Ramsden 1991). This highlights the importance of understanding not only the external elements of the learning context but also how students interpret and respond to these elements in shaping their academic experiences (Guo et al. 2023).

Scholars have long devoted attention to exploring the educational context (Richardson et al. 1999), learning assessments (Thomas and Bain 1984), and teaching approaches (Kember and Kwan 2000), as well as their impact on students’ learning strategies. In 1981, Ramsden and Entwistle (1981) conducted a pioneering empirical study to investigate the connection between learning approaches and students’ perceptions of their learning environment. Notably, this work emphasized students’ perceptions of the learning environment rather than the environment itself. Building on this, Ramsden developed the Course Experience Questionnaire (CEQ) to assess students’ perceptions of the teaching context (Ramsden 1991). He found that students who adopted a deep approach to learning typically viewed the teaching quality as excellent, the goals as clear, the workload and assessments as appropriate, and the level of academic freedom as satisfactory (Ramsden 1997). Since then, numerous researchers have explored the relationship between learners’ perceptions of the learning environment and their approach to deep learning. Many studies have investigated how various aspects of the learning environment influence deep learning strategies, consistently finding a coherent pattern of relationships between deep approaches and those environmental perceptions that foster deep learning among successful students (Trigwell and Prosser 1991; Wilson et al. 1997; Diseth 2007; Karagiannopoulou and Christodoulides 2005; Guo et al. 2017).

### 2.4. The Mediation Role of Achievement Goals

Achievement goals, as inherent cognitive orientations, reflect individuals’ underlying cognitive beliefs, purposes, and intentions when engaging in specific activities. Rooted in goal-setting and self-regulation theories, achievement goals represent a dynamic framework through which individuals interpret, approach, and respond to academic and situational demands (Dweck 1986; Elliot 1999). This cognitive orientation is not only shaped by individual predispositions but is also influenced by contextual and environmental factors, such as perceived learning environments. Achievement goal orientations provide a lens to understand students’ motivational dynamics and their engagement in learning processes.

The psychological foundation of achievement goals is rooted in the interplay between intrinsic and extrinsic motivational processes and the alignment between individuals’ beliefs about their competence and the perceived demands of tasks (Nicholls 1984). Mastery goals, closely tied to intrinsic motivation, emphasize the pursuit of competence, self-improvement, and mastery of tasks. Conversely, performance goals are predominantly extrinsic in nature, focusing on demonstrating competence relative to others (Elliot and Church 1997). These goals are further differentiated into approach and avoidance dimensions, reflecting individuals’ aspirations to achieve positive outcomes or their efforts to avoid negative evaluations. This nuanced distinction underpins the theoretical framework of the 2 × 2 achievement goal orientation model proposed by Elliot and McGregor (2001), which incorporates mastery-approach goals (MAG), mastery-avoidance (MAV), performance-approach (PAP), and performance-avoidance (PAV) goals orientations.

Prior research strongly supports the mediating role of achievement goals in the relationship between the learning environment and students’ learning approaches. The learning environment shapes the achievement goals students adopt, which in turn influence their motivation, learning strategies, and academic outcomes (Church et al. 2001; Lau and Lee 2008). Supportive, autonomy-promoting environments, characterized by encouragement and constructive feedback, foster mastery-oriented goals focused on competence development and personal growth, and these achievement goals driven by intrinsic motivation and conceptual understanding are closely associated with students’ deep learning approaches (Baeten et al. 2010; Asikainen and Gijbels 2017; Biggs 1999; Fawzia and Karim 2024). Conversely, competitive or high-stakes environments tend to promote performance-oriented goals, which may lead to surface or strategic learning approaches, as students prioritize external rewards or meeting external standards (Elliot et al. 1999; Covington 2000; Elliot and McGregor 2001; Geitz et al. 2015). Thus, achievement goals mediate the impact of the learning environment on students’ learning approaches, linking contextual factors to the quality of learning outcomes.

## 3. The Current Study

The literature reviewed above highlights a significant limitation in existing research: the predominant reliance on cross-sectional designs, which provide only a snapshot of the relationships between learning environment perception, achievement goals, and learning approaches. While such studies offer valuable insights, they fail to account for the dynamic and evolving processes through which achievement goals mediate the impact of perceived learning environments on students’ deep learning approaches over time. To develop a more comprehensive understanding of these complex interactions, it is crucial to adopt a longitudinal perspective. This approach allows for the examination of how achievement goal orientations evolve, interact with changing learning environments, and ultimately influence students’ sustained engagement and academic outcomes. Furthermore, current research primarily emphasizes the mediating role of mastery and performance-oriented achievement goals between learning environment perceptions and learning approaches, while the roles of approach and avoidance-oriented achievement goals remain underexplored in this context.

To address this gap, the present study employs a three-wave longitudinal tracking survey and constructs a cross-lagged model to explore these relationships. Specifically, this study aims to examine whether four types of achievement goals, as a mediating variable, play a longitudinal mediating role between undergraduates’ perceptions of their learning environment and their adoption of deep learning approaches. By focusing on these temporal processes, this study seeks to offer valuable insights that can inform educational practices, helping to foster environments that more effectively promote a deep learning approach.

## 4. Method

### 4.1. Participants

This study was conducted in June 2021, targeting first-year students at a research-oriented university located in eastern China, known for its industry-oriented education and outstanding disciplinary advantages. As a high-level research university, this institution assumes the responsibility of cultivating top innovative talents through a dual-cultivation model. Top-notch undergraduate students, referred to as “honors students”, are those who receive additional quality enhancement education through honors colleges beyond the general professional courses provided by standard colleges. In contrast, ordinary undergraduate students, also known as “non-honors students”, do not receive such supplementary education and are subject to the regular academic curriculum. To ensure the diversity of the sample, we selected both top undergraduate students and ordinary undergraduate students, aiming to explore their academic experiences on the key variables of the study.

Cluster sampling was initially employed to select outstanding undergraduate students from the Honors College. Subsequently, each participant was requested to invite one ordinary undergraduate from the general professional colleges to participate in the survey. The second and third rounds of data collection were conducted in March 2022 and December 2022, respectively, involving the participants who had taken part in the first round of the survey. Although there was some attrition during the questionnaire collection process, the effective sample sizes for the three rounds were 306, 289, and 285, respectively. The total sample size across all three rounds was 260, comprising 135 top-notch undergraduate students and 125 ordinary undergraduate students. Chi-square and *t*-tests revealed no significant differences between the attrition and retained participants in terms of gender ratio (χ2(1) = 0.670, *p* > .05), major type (t(306) = −0.215, *p* > .05), and deep learning approaches in the initial assessment (t(306) = 0.099, *p* > .05). Therefore, the analysis sample in this study is considered representative.

### 4.2. Measures

Deep learning approach. The R-SPQ-2F scale developed by Biggs et al. (2001) was used to measure the deep learning approach, consisting of 10 items related to deep learning. The total score of the deep learning approach is obtained by adding the scores of deep learning motivation and deep learning strategies. All items were rated on a 5-point Likert scale, with 1 representing completely inconsistent or rarely consistent and 5 representing completely or almost completely consistent. A higher score indicates a higher level of deep learning approach. In this study, Cronbach’s α coefficients for the deep learning motivation dimension across the three measurements were 0.865, 0.898, and 0.941, respectively. For the deep learning strategies dimension, Cronbach’s α coefficients for the three measurements were 0.817, 0.873, and 0.923, respectively.

Achievement goal. The Achievement Goal Scale developed by Elliot and McGregor (2001) was used, consisting of 12 items divided into four dimensions: performance-approach goals, mastery-avoidance goals, mastery-approach goals, and performance-avoidance goals. All items were rated on a 5-point Likert scale. In this study, the Cronbach’s α coefficients for the three measurements were as follows: performance-approach goal: 0.897, 0.910, 0.944; mastery-avoidance goal: 0.938, 0.938, 0.957; mastery-approach goal: 0.924, 0.928, 0.955; and performance-avoidance goal: 0.869, 0.915, 0.938.

Perception of learning environment. Good Teaching Scale (GTS), Generic Skills Scale (GSS), and Overall Satisfaction Inventory (OSI) in the Course Experience Questionnaire (CEQ) were developed to measure the perceived learning environment (Byrne and Flood 2003). All 13 items were measured using a 5-point Likert scale, with higher scores indicating a positive perception of the learning environment. The overall satisfaction (OSI) is a single-item indicator representing students’ general evaluation of the course experience, and therefore factor analysis is not conducted for this measure. Cronbach’s α coefficients for the three measurements of the Good Teaching Scale (GTS) were 0.951, 0.950, and 0.954, respectively, and for the General Skills Scale (GSS), the Cronbach’s α coefficients were 0.963, 0.962, and 0.964, respectively.

The items of the aforementioned scales are described in Appendix A. Based on the study participants’ characteristics, a self-designed questionnaire was used to gather information on their personal background, college entrance examination situation, family background, and other information. Most of the items were multiple-choice questions, and the options for the multiple-choice questions were not right or wrong. Participants were asked to select the appropriate answer based on their actual situation. A small portion of the items were fill-in-the-blank questions, where students were required to enter the appropriate number on the blank line based on their actual situation.

### 4.3. Procedure

With the informed consent of the college administrators and the students themselves, the first wave of surveys was conducted in June 2021, targeting undergraduate students in their freshman year. In March 2022 and December 2022, invitations for the second and third waves of surveys were, respectively, sent to the undergraduate students who participated in the first wave. Prior to administering the survey, participants were informed that there were no right or wrong answers to the questionnaire items, and their responses would be kept completely confidential to ensure that they could answer according to their actual circumstances. During the survey administration process, electronic questionnaires were distributed online by the monitors of each class, using unified instructions. The questionnaire completion time was approximately 8–10 min. After completing the questionnaire, each participant received a certain reward.

### 4.4. Data Analysis

The analysis was conducted using SPSS 23.0 and Mplus 8.0 in this study. As the data collected from undergraduate students were all self-reported, a common method bias test was conducted using SPSS 23.0. Furthermore, in the process of testing the longitudinal mediation effects, descriptive statistics and correlation analysis of the variables were initially performed using SPSS 23.0. Finally, a fixed-effects cross-lagged panel model (CLPM) was constructed using Mplus 8.0.

The cross-lagged panel model (CLPM), as a statistical model used to analyze the interrelationships between variables in longitudinal data, can effectively determine causal relationships between variables and evaluate the mutual influence effects between them. By considering the measurement results of variables at different time points, the CLPM can capture the dynamic changes between variables and provide information on how these variables influence each other. By analyzing the temporal evolution of variables, the CLPM can reveal both long-term and short-term dynamics between variables and how they interact with each other. This facilitates a deeper understanding of the developmental trajectory of variables and the identification of key factors influencing variable changes. Additionally, the CLPM can utilize known variable information to predict future variable states. By establishing a relationship model between variables, the CLPM can infer variable values at a certain time point, thus providing predictions for future development. In summary, this study effectively explores the interactive relationships and dynamic changes between variables that influence undergraduate students’ deep learning styles by constructing a cross-lagged panel model. It holds practical implications for higher education institutions in formulating educational programs and implementing teaching interventions.

**Figure 1:**
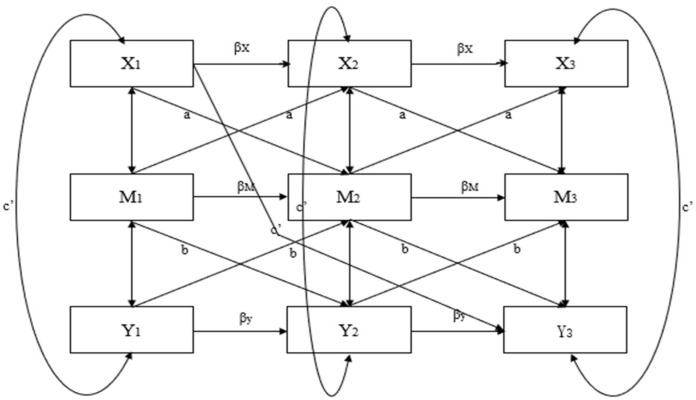
Second-order lagged CLPM.

Given that this study includes three rounds of longitudinal tracking data, a second-order lagged CLPM was selected as the longitudinal mediation model. Assuming that the variables have already been standardized, the longitudinal mediation effects of the second-order lagged CLPM (see Figure 1) can be represented as follows:

X_t+1_ = β_x_X_t_ + ε_x(t+1)_ (1)

M_t+1_ = β_M_M_t_ + aX_t_+ε_M(t+1)_ (2)

Y_t+2_ = β_Y_Y_t+1_ + bM_t+1_ + c′X_t_+ε_Y(t+2)_ (3)

Each variable in the model depends on both the previous measurement levels of the antecedent variables and the previous measurement levels of the variable itself. The regression of each variable on the antecedent variables is referred to as lag effects (represented by coefficients a, b, and c′). The effects of variables at different time points are known as autoregressive effects (represented by coefficients β_X_, β_M_, and β_Y_, respectively). The coefficient ab represents the longitudinal mediation effect, c′ represents the direct effect, and the subscripts t, t + 1, and t + 2 indicate the time points of repeated measurements (Fang et al. 2021). 

To simplify the model and improve model fit, item parceling was performed on the items, and random parceling was used for all variables. Full Information Maximum Likelihood (FIML) estimation was employed to handle non-normal data and missing values. The mediation effects of the latent variable model were tested using a bias-corrected bootstrap method with 5000 resamples.

## 5. Results

### 5.1. Common Method Bias Test

Since the sample data in this study were obtained from self-reports by students, a common method bias test was conducted. Harman’s single-factor test was performed on the three waves of data, and the results revealed that the variance explained by the first factor was 16.81%, 18.41%, and 18.85%, respectively, all of which were below the critical threshold of 40%. Therefore, there was no evident issue of common method bias in the data of this study.

### 5.2. Descriptive Statistics and Correlation Analysis

This study utilized the achievement goal inventory developed by Elliot and McGregor, which classifies learners’ achievement goal orientations into four types: performance-approach goals, mastery-avoidance goals, mastery-approach goals, and performance-avoidance goals. Descriptive statistics and correlation coefficients between undergraduate students’ perceived learning environment, the four types of achievement goal orientations, and deep learning approaches are presented in Table 1, Table 2, Table 3 and Table 4.

Table 1 reveals the temporal stability of undergraduate students’ perceived learning environment, mastery-approach goals, and deep learning approaches. Significant positive correlations were observed between the learning environment perception at T1 and T2, mastery-approach goals at T2, deep learning approach at T2, learning environment perception at T3, mastery-approach goals at T3, and deep learning approach at T3. These findings indicate a significant positive association between undergraduate students’ perceived learning environment, mastery-approach goals, and deep learning approaches across time.

As shown in Table 2, there are significant positive concurrent correlations between learning environment perception, mastery-avoidance goal orientation, and deep learning approach among undergraduates at T1, T2, and T3. Specifically, the learning environment perception of undergraduates at T1 is significantly positively correlated with their mastery-avoidance goal orientations and deep learning approaches at T1, and the same pattern is observed at T2 and T3. Furthermore, the lagged relationships between the same variables vary across different stages. Specifically, the relationship between mastery-avoidance goal orientation at T3 and mastery-avoidance goal orientation at T1 and T2 is not significant, while the relationship between learning environment perception at T3 and learning environment perception at T1 and T2 and the relationship between deep learning approach at T3 and deep learning approach at T1 and T2 is significant. In addition, there are significant lagged correlations between the three variables. Specifically, learning environment perception at T1 is significantly correlated with mastery-avoidance goal orientation at T2, and mastery-avoidance goal orientation at T2 significantly predicts a deep learning approach at T3.

As shown in Table 3, there were concurrent significant positive correlations between undergraduate students’ perception of the learning environment, performance-approach goal orientation, and deep learning approach at the T1, T2, and T3 stages. Specifically, T1 perception of the learning environment was significantly positively correlated with T1 performance-approach goal orientation and T1 deep learning approach. Similarly, T2 perception of the learning environment was significantly positively correlated with T2 performance-approach goal orientation and T2 deep learning approach. Furthermore, T3 perception of the learning environment was significantly positively correlated with T3 performance-approach goal orientation and T3 deep learning approach. Additionally, there were consistent lagged correlations between the same variables across different stages. For instance, T1 perception of the learning environment was significantly positively correlated with T2 perception of the learning environment and T3 perception of the learning environment. Likewise, performance-approach goal orientation at T1, T2, and T3 showed significant positive lagged correlations. Similarly, the deep learning approach at T1, T2, and T3 exhibited significant positive lagged correlations. Moreover, there were significant lagged correlations between the three variables as well. Specifically, T1 perception of the learning environment was significantly correlated with T2 performance-approach goal orientation, and T2 performance-approach goal orientation could significantly predict the T3 deep learning approach.

As presented in Table 4, the concurrent correlations between undergraduate students’ perception of the learning environment, performance-avoidance goal orientation, and deep learning approach at the T1, T2, and T3 stages were not consistently significant. Specifically, there was no significant correlation between T1 perception of the learning environment and T1 performance-avoidance goal orientation. T2 perception of the learning environment showed a significant negative correlation with T2 performance-avoidance goal orientation and T2 deep learning approach. Similarly, T3 perception of the learning environment exhibited a significant negative correlation with T3 performance-avoidance goal orientation and T3 deep learning approach. Furthermore, consistent lagged correlations between the same variables across different stages were not observed. T1 perception of the learning environment, T2 perception of the learning environment, and T3 perception of the learning environment were significantly positively correlated with T1 deep learning approach, T2 deep learning approach, and T3 deep learning approach. However, there was no significant relationship between T1 performance-avoidance goal orientation and T2 performance-avoidance goal orientation. Moreover, there were significant negative lagged correlations between the three variables. Specifically, T1 perception of the learning environment was significantly negatively correlated with T2 performance-avoidance goal orientation, and T2 performance-avoidance goal orientation could significantly negatively predict the T3 deep learning approach.

### 5.3. Testing the Longitudinal Mediation Model

This study examines undergraduate students’ perception of the learning environment as an independent variable, the deep learning approach as a dependent variable, and the four types of achievement goal orientations as mediating variables. By constructing a cross-lagged model that incorporates different types of achievement goal orientations, the study aims to explore whether these orientations mediate the relationship between independent and dependent variables in a longitudinal investigation. If significant mediation effects are found, further analysis will explore the differences in the mediation effects between the relevant variables. If the mediation effects are not significant, the study will provide a detailed explanation of the relationships and causal pathways between different types of achievement goal orientations and variables at different stages of learning.

**Figure 2:**
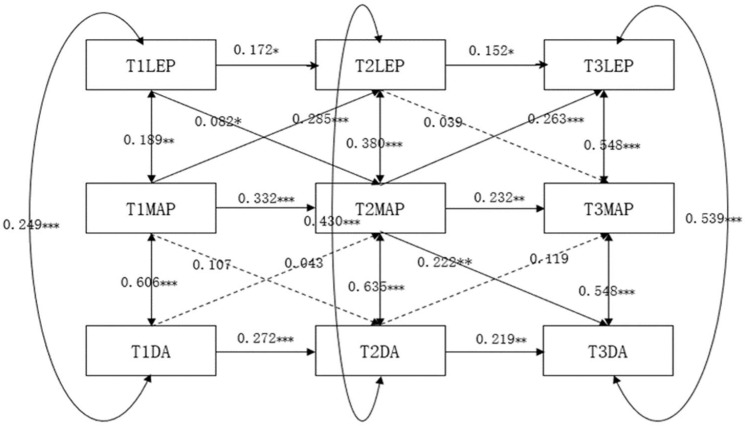
The cross-lagged regression analysis of learning environment perception, mastery-approach goals, and deep learning approach.

The cross-lagged regression models of learning environment perception, mastery-approach goals, and undergraduate students’ deep learning approach are presented in Figure 2. After gradually removing nonsignificant paths, the model shows a good fit with χ2/df = 2.285, RMSEA = 0.040, CFI = 0.974, TLI = 0.935, and SRMR = 0.059. Constructing a longitudinal mediation model with mastery-approach goals as the mediating variable, the results demonstrate that mastery-approach goals not only mediate the concurrent relationship between undergraduate students’ perception of the learning environment and deep learning approach but also play a significant lagged mediation role in the longitudinal development process between learning environment perception and deep learning approach. Specifically, T1mastery-approach goals mediate the relationship between T1 perception of the learning environment and T1 deep learning approach, T2 mastery-approach goals mediate the relationship between T2 perception of the learning environment and T2 deep learning approach, and T3 mastery-approach goals mediate the relationship between T3 perception of the learning environment and T3 deep learning approach.

Furthermore, the longitudinal effects of mastery-approach goals on undergraduate students’ perception of the learning environment and deep learning approach remain significant. That is, T1 perception of the learning environment significantly influences T2 mastery-approach goals, which in turn affects T3 deep learning approach. In conclusion, mastery-approach goals are a longitudinal mediating factor between undergraduates’ perception of the learning environment and their deep learning approach. The results of the bootstrap analysis indicated that mastery-approach goals at Time 2 (ab = 0.582, *p* = 0.000, 95% CI = 0.013~0.103) had a significant mediating effect on the prediction of learning environment perception (T1) and deep learning approach (T3). The mediated effect accounted for 31.328% of the total effect.

**Figure 3:**
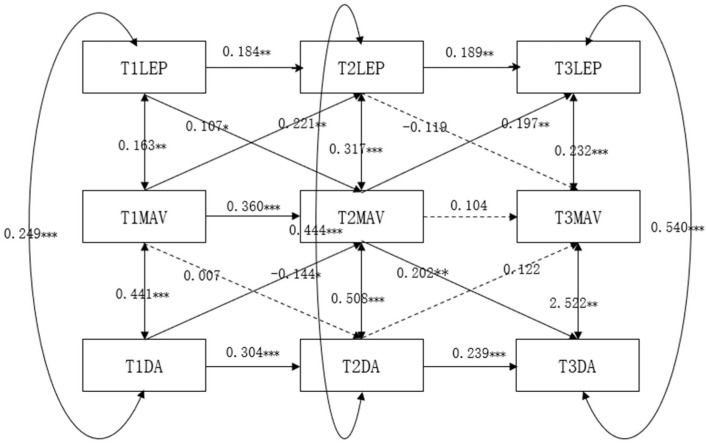
The cross-lagged regression analysis of learning environment perception, mastery-avoidance goals, and deep learning approach.

The cross-lagged regression model of learning environment perception, mastery-avoidance goals, and deep learning approach among undergraduate students is presented in Figure 3. After gradually eliminating nonsignificant paths, the model fit indices indicated a good fit: χ2/df = 3.260, RMSEA = 0.043, CFI = 0.935, TLI = 0.835, SRMR = 0.065. Constructing a longitudinal mediation model with undergraduate students’ mastery-avoidance goals as the mediating variable, the results revealed that mastery-avoidance goals not only played a contemporaneous significant mediating role in the relationship between learning environment perception and deep learning approach among college students but also demonstrated a significant longitudinal mediating effect in the developmental process of learning environment perception and deep learning approach. Specifically, mastery-avoidance goals mediated the relationship between learning environment perception and deep learning approach at T1; mastery-avoidance goals mediated the relationship between learning environment perception and deep learning approach at T2; mastery-avoidance goals mediated the relationship between learning environment perception and deep learning approach at T3. Furthermore, the longitudinal effects of mastery-avoidance goals in the process of learning environment perception and deep learning approach among college students remained significant, indicating that T1 learning environment perception significantly influenced T2 mastery-avoidance goals, which in turn had an impact on undergraduate students’ T3 deep learning approach. In summary, mastery-avoidance goals play a longitudinal mediating role between undergraduates’ perception of the learning environment and their deep learning approach. The results of the bootstrap analysis revealed that mastery-avoidance goals at Time 2 (ab = 0.482, *p* = 0.000, 95% CI = 0.008~0.094) had a significant mediating effect on the prediction of learning environment perception (T1) and deep learning approach (T3). The mediated effect accounted for 25.926% of the total effect.

**Figure 4:**
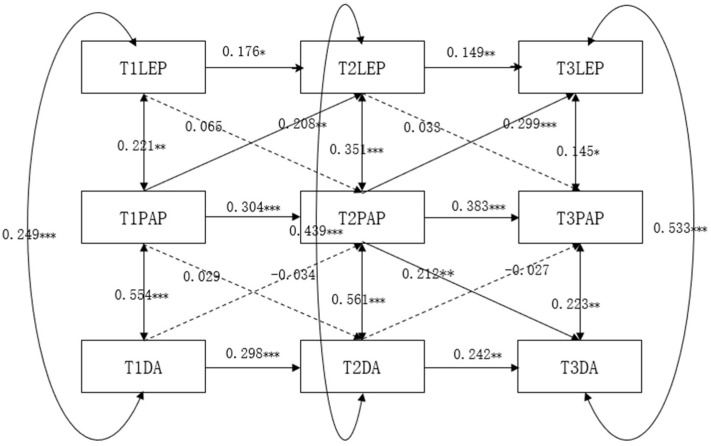
The cross-lagged regression analysis of learning environment perception, performance-approach goals, and deep learning approach.

The cross-lagged regression model of learning environment perception, performance-approach goals, and deep learning approach among undergraduate students is presented in Figure 4. After gradually eliminating nonsignificant paths, the model fit indices indicated a good fit: χ2/df = 2.957, RMSEA = 0.026, CFI = 0.948, TLI = 0.869, SRMR = 0.060. Constructing a longitudinal mediation model with undergraduate students’ performance-approach goals as the mediating variable, the results revealed that performance-approach goals only played a contemporaneous significant mediating role in the relationship between learning environment perception and deep learning approach among college students and did not exhibit a significant longitudinal mediating effect. Specifically, at T1, performance-approach goals mediated the relationship between learning environment perception and deep learning approach; performance-approach goals mediated the relationship between learning environment perception and deep learning approach at T2; performance-approach goals mediated the relationship between learning environment perception and deep learning approach at T3. However, the longitudinal effects of performance-approach goals in the process of learning environment perception and deep learning approach among college students were not significant, indicating that T1 learning environment perception did not significantly influence T2 performance-approach goals, which in turn had no impact on the T3 deep learning approach.

**Figure 5:**
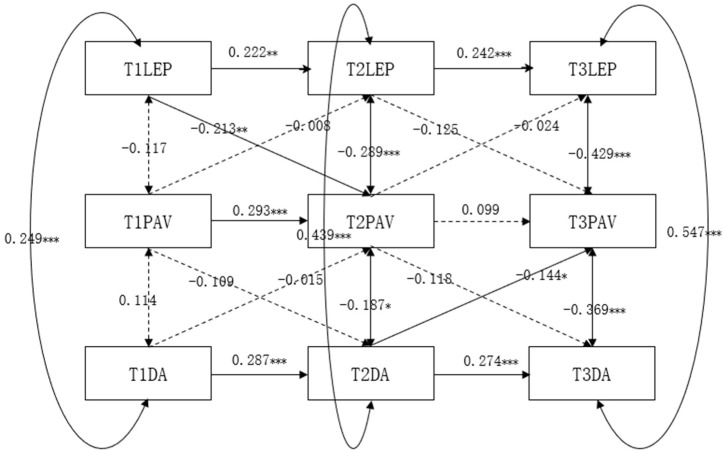
The cross-lagged regression analysis of learning environment perception, performance-avoidance goals, and deep learning approach.

The cross-lagged regression model of learning environment perception, performance-avoidance goals, and deep learning approach among undergraduate students is presented in Figure 5. After gradually eliminating nonsignificant paths, the model fit indices indicated a good fit: χ2/df = 4.362, RMSEA = 0.044, CFI = 0.906, TLI = 0.862, SRMR = 0.060. Constructing a longitudinal mediation model with undergraduate students’ performance-avoidance goals as the mediating variable, the results revealed that performance-avoidance goals only played a partial contemporaneous significant mediating role in the relationship between learning environment perception and deep learning approach among college students and did not exhibit a significant longitudinal mediating effect. Specifically, at T2, performance-avoidance goals mediated the relationship between learning environment perception and deep learning approach; at T3, performance-avoidance goals mediated the relationship between learning environment perception and deep learning approach; however, at T1, the mediating effect of performance-avoidance goals between learning environment perception and deep learning approach was not significant. Furthermore, the longitudinal effects of performance-avoidance goals in the process of learning environment perception and deep learning approach among college students were not significant, indicating that T1 learning environment perception did not significantly influence T2 performance-avoidance goals, which in turn had no impact on the T3 deep learning approach.

## 6. Discussion

This study investigates the longitudinal interactive relationships between undergraduates’ perception of the learning environment, achievement goal orientation, and deep learning approach through a comprehensive 18-month, three-wave tracking survey. Previous studies on the relationship between undergraduates’ perception of the learning environment and deep learning approach have not thoroughly explored the longitudinal mediating role of achievement goals. Therefore, it could provide additional longitudinal evidence to prove the vertical interaction between learning environment perception, achievement goal, and deep learning approach.

### 6.1. Longitudinal Mediation Role of Different Types of Achievement Goals

The results of this study show that different types of achievement goals have varying longitudinal mediation effects between undergraduates’ perception of the learning environment and their deep learning approach. Specifically, both performance-approach goals and performance-avoidance goals do not serve as longitudinal mediators in the influence of undergraduates’ perception of the learning environment on their deep learning approach. However, mastery-approach goals and mastery-avoidance goals can partially mediate the longitudinal impact of undergraduates’ perception of the learning environment on their deep learning approach. These findings align with previous cross-sectional studies (Elliot and Church 1997), which suggest that mastery-approach goals and mastery-avoidance goals can stimulate learners’ intrinsic motivation, whereas performance-approach goals are often associated with improved grades, and performance-avoidance goals tend to decrease intrinsic motivation and learning performance.

In addition, the results of this study show that, compared to mastery-avoidance goals, mastery-approach goals have a larger proportion of the mediating effect. This indicates that in the developmental process of the influence of undergraduates’ perception of the learning environment on their deep learning approach, mastery-approach goals play a more prominent longitudinal mediating role. Mastery-approach goals reflect individuals’ desire for self-improvement, while mastery-avoidance goals reflect individuals’ desire to avoid performing worse than expected (Ortiz et al. 2023). Previous research has validated that mastery-avoidance goals and mastery-approach goals may have different effects on various aspects of individuals’ psychological functioning, such as task performance, well-being, and attention (Elliot 2008; Van Yperen et al. 2009). Individuals who adopt mastery-approach goals strive to maximize their potential and possess a strong belief in their ability to succeed, while those with a mastery-avoidance orientation tend to harbor fear and apprehension about their capacity to master a task or capitalize on the given situation (Stoeber et al. 2008).

### 6.2. Longitudinal Predictive Pathway from the Perception of Learning Environment to Deep Learning Approach

The development of a deep learning approach relies not only on external environmental support but also on the intrinsic motivation of learners generated through their perception of the external learning environment and its influence on their learning behavior. The mastery goals formed by learners based on their perception of the external learning environment can play a positive role in the development of their deep learning approach. This study employed a cross-lagged model to examine the longitudinal predictive pathways of perceived learning environment on deep learning approaches, with four different types of achievement goal orientations as mediating variables. The results revealed two mediating pathways, namely the mastery-approach goal orientation and mastery-avoidance goal orientation, in the influence process of perceived learning environment on deep learning approaches among undergraduate students.

Firstly, positive feedback from the learning environment transforms students’ beliefs about goals, abilities, and success during task completion (Pintrich 2000). Under the influence of mastery-approach goals, learners significantly enhance their deep learning levels. Mastery-approach goals awaken intrinsic motivation, which fosters genuine interest in the learning process and promotes deep and meaningful learning (Elliot and Church 1997). Students driven by mastery-approach goals prioritize understanding, persistence, and improvement over mere performance outcomes (Van Yperen et al. 2009). They are more likely to employ strategies such as critical thinking and problem-solving while valuing the process of learning itself. Moreover, mastery-approach goals encourage autonomy and self-regulation in learning, enabling students to connect new knowledge with prior understanding and apply it across diverse contexts. Existing studies consistently demonstrate the positive effects of mastery-approach goals on engagement, self-regulated learning, and academic performance (Stoeber et al. 2008; Elliot 2008).

Secondly, under the perception of the external learning environment, undergraduate students develop mastery-avoidance goals, which in turn lead to an improvement in the level of deep learning approaches. The importance and role of mastery-avoidance goals in educational and learning contexts are worth contemplating. Although traditional research has often associated mastery-avoidance goals with negative learning outcomes (Conroy and Elliot 2004; Conroy et al. 2003; Stoeber et al. 2008), these goals may also promote reflective learning. For instance, mastery-avoidance goals could encourage students to critically evaluate their limitations, seek feedback, and focus on growth opportunities (Morris and Kavussanu 2008).

### 6.3. Implications

Based on the findings of this study, several practical implications can be drawn for educators and administrators aiming to enhance students’ deep learning engagement and academic success.

First, it is essential to create learning environments that emphasize personal growth, mastery, and self-improvement rather than competition. Promoting mastery-approach goals, which focus on content mastery and self-improvement, can significantly enhance students’ intrinsic motivation. This can be achieved through curriculum design, teacher feedback, and fostering a classroom culture that encourages deep engagement with learning. Given that mastery-approach goals play a strong mediating role in the relationship between learning environment perception and deep learning approaches, encouraging this goal orientation is crucial for improving academic outcomes.

Second, while mastery-avoidance goals are often associated with negative outcomes, they can also have potential benefits if properly leveraged. Institutions should design interventions that help students transform their fear of failure into a positive driving force, encouraging resilience and reflective thinking. Providing guidance through workshops on goal setting and self-regulation can assist students in setting realistic and meaningful objectives, helping them overcome challenges and engage in more focused and constructive learning.

Finally, it is important to tailor interventions to address the diversity of students’ achievement goal orientations. By recognizing the different ways in which mastery-approach and mastery-avoidance goals can impact learning, institutions can help students harness the positive aspects of both goal types. This approach not only supports individual development but also promotes sustained academic engagement and success.

### 6.4. Limitations and Future Research

Some limitations of this study should be addressed. Firstly, the relatively small sample size may limit the generalizability of the findings. To address this, we suggest future research could benefit from a larger and more diverse sample size, as this would enhance the robustness and applicability of the conclusions across different populations and contexts. Secondly, our research sample was derived from a prestigious industry-specific research university in China. Subsequent studies could encompass diverse types of universities for survey administration, aiming to elucidate distinctions among various institutional categories and enhance the generalizability of research conclusions. Thirdly, this study conducted three rounds of tracking surveys. However, these three survey rounds remain insufficient to unveil the comprehensive developmental trajectory of undergraduate students’ learning environment perception, achievement goals, and deep learning approaches during their university years. Future research could further extend the tracking duration to provide a more comprehensive understanding.

## Figures and Tables

**Table 1:** Descriptive statistics and correlation coefficients of learning environment perception, mastery-approach goals, and deep learning approach.

	S	MD	1	2	3	4	5	6	7	8	9
1. T1LEP	3.265	0.902	1								
2. T2LEP	3.469	0.980	0.243 ***	1							
3. T3LEP	3.446	1.143	0.124 *	0.316 ***	1						
4. T1MAP	3.786	0.926	0.189 **	0.317 ***	0.206 **	1					
5. T2MAP	3.596	1.068	0.160 **	0.450 ***	0.330 ***	0.321 ***	1				
6. T3MAP	3.500	1.193	0.173 **	0.248 ***	0.613 ***	0.200 **	0.324 ***	1			
7. T1DA	33.350	8.330	0.249 ***	0.304 ***	0.188 **	0.606 ***	0.213 **	0.239 ***	1		
8. T2DA	34.010	9.151	0.127 *	0.493 ***	0.401 ***	0.269 ***	0.649 ***	0.373 ***	0.371 ***	1	
9. T3DA	36.570	9.653	0.174 **	0.289 ***	0.621 ***	0.225 ***	0.359 ***	0.622 ***	0.263 ***	0.441 ***	1

Notes: T1 represents Time 1, T2 represents Time 2, T3 represents Time 3, LEP stands for learning environment perception, DA stands for deep learning approach, MAP stands for mastery-approach goals, and *** *p* < 0.001, ** *p* < 0.05, and * *p* < 0.1 are indicated in all tables and figures below.

**Table 2:** Descriptive statistics and correlation coefficients of learning environment perception, mastery-avoidance goals, and deep learning approach.

	M	SD	1	2	3	4	5	6	7	8	9
1. T1LEP	3.265	0.902	1								
2. T2LEP	3.469	0.980	0.243 ***	1							
3. T3LEP	3.446	1.143	0.124 *	0.316 ***	1						
4. T1MAV	3.537	1.065	0.163 **	0.250 ***	0.096	1					
5. T2MAV	3.350	1.131	0.157 *	0.360 ***	0.262 ***	0.315 ***	1				
6. T3MAV	3.406	1.179	−0.038	0.007	0.258 ***	−0.014	0.118	1			
7. T1DA	33.350	8.330	0.249 ***	0.304 ***	0.188 **	0.441 ***	0.092	−0.014	1		
8. T2DA	34.010	9.151	0.127 *	0.493 ***	0.401 ***	0.138 *	0.471 ***	0.166 **	0.371 **	1	
9. T3DA	36.570	9.653	0.174 **	0.289 ***	0.621 ***	0.127 *	0.307 ***	0.286 ***	0.263 ***	0.441 ***	1

Notes: MAV stands for mastery-avoidance goals.

**Table 3:** Descriptive statistics and correlation coefficients of learning environment perception, performance-approach goals, and deep learning approach.

	M	SD	1	2	3	4	5	6	7	8	9
1. T1LEP	3.265	0.902	1								
2. T2LEP	3.469	0.980	0.243 ***	1							
3. T3LEP	3.446	1.143	0.124 *	0.316 ***	1						
4. T1PAP	3.683	0.996	0.221 ***	0.246 ***	0.252 ***	1					
5. T2PAP	3.526	1.105	0.151 *	0.402 ***	0.357 ***	0.299 ***	1				
6. T3PAP	3.568	1.105	0.095	0.194 **	0.262 ***	0.200 **	0.381 ***	1			
7. T1DA	33.350	8.330	0.249 ***	0.304 ***	0.188 **	0.554 ***	0.202 **	0.085	1		
8. T2DA	34.010	9.151	0.127 *	0.493 ***	0.401 ***	0.191 **	0.566 ***	0.229 ***	0.371 ***	1	
9. T3DA	36.570	9.653	0.174 **	0.289 ***	0.621 ***	0.218 ***	0.343 ***	0.319 ***	0.263 ***	0.441 ***	1

Notes: PAP stands for performance-approach goals.

**Table 4:** Descriptive statistics and correlation coefficients of learning environment perception, performance-avoidance goals, and deep learning approach.

	M	SD	1	2	3	4	5	6	7	8	9
1. T1LEP	3.265	0.902	1								
2. T2LEP	3.469	0.980	0.243 ***	1							
3. T3LEP	3.446	1.143	0.124 *	0.316 ***	1						
4. T1PAV	2.562	1.016	−0.117	−0.034	−0.003	1					
5. T2PAV	2.571	1.185	−0.259 ***	−0.327 ***	−0.102	0.316 ***	1				
6. T3PAV	2.673	1.191	−0.114	−0.271 ***	−0.500 ***	0.015	0.171 **	1			
7. T1DA	33.350	8.330	0.249 ***	0.304 ***	0.188 **	0.114	−0.103	−0.140 *	1		
8. T2DA	34.010	9.151	0.127 *	0.493 ***	0.401 ***	−0.074	−0.233 ***	−0.345 ***	0.371 ***	1	
9. T3DA	36.570	9.653	0.174 **	0.289 ***	0.621 ***	0.007	−0.176 **	−0.462 ***	0.263 ***	0.441 ***	1

Notes: PAV stands for performance-avoidance goals.

## Data Availability

The data presented in this study are available on request from the corresponding authors due to privacy and ethical restrictions.

## Appendix A

Deep learning approach:

1. I find that, at times, studying gives me a feeling of deep personal satisfaction.
2. I find that I have to do enough work on a topic so that I can form my own conclusions before I am satisfied.
3. I feel that virtually any topic can be highly interesting once I get into it.
4. I find most new topics interesting and often spend extra time trying to obtain more information about them.
5. I find that studying academic topics can, at times, be as exciting as a good novel or movie.
6. I test myself on important topics until I understand them completely.
7. I work hard at my studies because I find the material interesting.
8. I spend a lot of my free time finding out more about interesting topics that have been discussed in different classes.
9. I come to most classes with questions in mind that I want answered.
10. I make a point of looking at most of the suggested readings that go with the lectures.

Achievement goal:

1. It is important for me to do better than other students.
2. It is important for me to do well compared to others in this class.
3. My goal in this class is to get a better grade than most of the other students.
4. I worry that I may not learn all that I possibly could in this class.
5. Sometimes, I’m afraid that I may not understand the content of this class as thoroughly as I’d like.
6. I am often concerned that I may not learn all that there is to learn in this class.
7. I want to learn as much as possible from this class.
8. It is important for me to understand the content of this course as thoroughly as possible.
9. I desire to completely master the material presented in this class.
10. I just want to avoid doing poorly in this class.
11. My goal in this class is to avoid performing poorly.
12. My fear of performing poorly in this class is often what motivates me.

Perception of learning environment:

1. The teaching staff of this course motivated me to do my best work.
2. The staff put a lot of time into commenting on my work.
3. The staff made a real effort to understand difficulties I might be having with my work.
4. The teaching staff normally gave me helpful feedback on how I was going.
5. My lecturers were extremely good at explaining things.
6. The teaching staff worked hard to make their subjects interesting.
7. The course developed my problem-solving skills.
8. The course sharpened my analytical skills.
9. The course helped me develop my ability to work as a team member.
10. As a result of my course, I feel confident about tackling unfamiliar problems.
11. The course improved my skills in written communication.
12. My course helped me to develop the ability to plan my own work.
13. Overall, I am satisfied with the quality of the courses.
